# Sex differences in clinical characteristics and risk factors for disease severity of hospitalized patients with COVID‐19

**DOI:** 10.1002/mco2.66

**Published:** 2021-04-08

**Authors:** Jing‐Jing Wang, Yun‐Juan Su, Qi Wang, Ying Cao, Ai‐Bin Wang, Rui Ding, Wen Xie

**Affiliations:** ^1^ Center of Liver Diseases, Beijing Ditan Hospital Capital Medical University Beijing China; ^2^ Department of Cardiology, Beijing Ditan Hospital Capital Medical University Beijing China; ^3^ Infectious Diseases Diagnostic, Therapeutic and Research Centre, Beijing Ditan Hospital Capital Medical University Beijing China

**Keywords:** COVID‐19, disease severity, risk factors, SARS‐CoV‐2, sex differences

## Abstract

Recent studies reported sex differences in patients with coronavirus disease‐2019 (COVID‐19). We aim to analyze sex differences in clinical characteristics and risk factors for disease severity of hospitalized patients with COVID‐19 in Beijing. All adults (185 cases) diagnosed with COVID‐19 and admitted to Beijing Ditan Hospital, Capital Medical University were included in samples. The median age of all patients was 41 years. The mean body mass index (BMI) of males was relatively higher compared to females (*p *< 0.001). The proportion of male patients with coronary heart disease (CHD), nonalcoholic fatty liver disease (NAFLD), history of smoking and drinking was higher than females. Male patients developed more clinical symptoms, obtained more abnormal laboratory test results, while they were less aware of care‐seeking than female patients. There were no significant differences in clinical complications and outcomes between two groups. Age (odds ratio [OR]: 1.082; 95% confidence interval [CI]: 1.034–1.132; *p *= 0.001) and BMI (OR: 1.237; 95% CI: 1.041–1.47; *p *= 0.016) were considered risk factors for refractory pneumonia in multivariate regression analysis. The findings of the current study showed that SARS‐CoV‐2 was more likely to affect older males with comorbidities. Further researches into factors underlying obesity and disease severity may provide mechanistic insight into COVID‐19 development.

## Graphical abstract

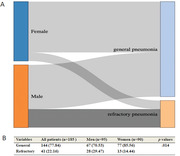

**Abbreviations**
AKI: acute kidney injury
ARDS: acute respiratory distress syndrome
AST: aspartate aminotransferase
BMI: body mass index
CHD: coronary heart disease
CI: confidence interval
CK: creatine kinase
COVID‐19: coronavirus disease‐2019
CRP: c‐reactive protein
CT: computerized tomography
DBP: diastolic blood pressure
DIC: diffuse intravascular coagulation
EMR: electronic medical record
FIB: fibrinogen
HT: hypertension
MERS: Middle East respiratory syndrome
NAFLD: nonalcoholic fatty liver disease
OR: odds ratio
SARS: severe acute respiratory syndrome
SARS‐CoV‐2: severe acute respiratory syndrome coronavirus 2
SBP: systolic blood pressure
T2DM: type 2 diabetes mellitus

## 1 INTRODUCTION

Coronavirus disease‐2019 (COVID‐19) is an emerging respiratory infectious disease caused by the severe acute respiratory syndrome coronavirus 2 (SARS‐CoV‐2) and triggered a global pandemic which has not been under control yet.^1^, ^2^ Even the situation has been curbed in some countries as of July 11, there are still 12,322,395 cases diagnosed and 556,335 deaths confirmed around the world. Previous reports have reported that there existed a sex imbalance based on confirmed cases and case fatality rate of COVID‐19.^3^ Notably, the roles and significance of sex are often neglected in studies of infectious disease.^4^ As studied, hormone difference alters the host response to infection disease, and sex‐biasing differences influence host pathogen interactions.^5^ Previous studies have reported that European and American had higher percentage of male patients with COVID‐19 were far worse than females.^6^ More than that, male sex, older age, underlying disease such as type 2 diabetes mellitus (T2DM), hypertension (HT), obesity, coronary heart disease (CHD) are risk factors associated with worse outcomes.^7^ The health status of Beijing residents has reached the level of high‐income countries and regions, and the health status of women is better than that of men. Based on recent reports, the prevalence of overweight was the highest in Beijing among Chinese adults published in 2019, the prevalence of overweight was the highest in Beijing.^8^, ^9^ The differences in the health status of men and women might contribute to the sex differences in COVID‐19, and the characteristics of COVID‐19 may be different from those of other cities as well. However, sex differences associated with clinical characteristics, severity and mortality of COVID‐19 in Beijing have not been well described yet. We summarized the clinical characteristics, comorbid conditions, severity and outcomes of 185 hospitalized patients in order to investigate whether sex bias is associated with the clinical characteristics and early outcomes of the patients who were diagnosed with COVID‐19 in Beijing and to assess potential risk factors on patients with severe COVID‐19 at admission.

## 2 METHODS

### 2.1 Study design and participants

From January 13, 2019 to March 19, 2020, a total of 185 adults with COVID‐19 were diagnosed and admitted if tested positive for respiratory symptoms, showed typical chest imaging findings, and tested positive SARS‐CoV‐2 test results of pharyngeal swab specimens in Beijing Ditan Hospital. Data collected through the electronic medical record system included baseline demographic information (age, sex, body mass index [BMI], smoking history, and alcohol consumption), underlying disease (CHD, HT, T2DM, respiratory disease, and nonalcoholic fatty liver disease [NAFLD]), symptoms (fever, headache, fatigue, cough, sore throat, cough, expectoration, shortness of breath, nausea/vomiting, myalgia/arthralgia, diarrhea), vital signs (indoor air oxygen saturation, the highest temperature, heart rate [HR], systolic blood pressure [SBP],diastolic blood pressure [DBP]), laboratory data of the first 24‐h hospital stay (leukocyte count, neutrophil count, lymphocyte count, neutrophil/lymphocyte ratio (NLR), hemoglobin, platelet count, creatinine, AST, alanine aminotransferase (ALT), troponin T, bilirubin, albumin, creatine kinase (CK), lactate dehydrogenase, d‐dimer, fibrinogen (FIB), CRP, procalcitonin, serum amyloid A (SAA)), computerized tomography (CT), clinical complications including acute respiratory distress syndrome (ARDS), acute kidney injury (AKI) or diffuse intravascular coagulation (DIC), septic shock, co‐infection, myocardial damage and final diagnosis including remained in hospital, discharge, death, and re‐admission. All data were analyzed independently by three researchers and obtained in accordance with standard biosafety and institutional safety procedures during the observation period of this study. The severity of the patient with COVID‐19 is determined based on the diagnostic and treatment guideline for SARS‐CoV‐2 issued by Chinese National Health Committee (version 7).^10^

### 2.2 Statistical analysis

The normality of continuous variables’ distribution was tested by one‐sample Kolmogorov‐Smirnov test. Continuous variables with normal distribution were presented as mean ± standard deviation (SD). Mean of two continuous normally distributed variables were compared using independent samples Student's test; non‐normal variables were reported as median (interquartile range [IQR]). Mann‐Whitney U test and Kruskal‐Wallis test were used, respectively, to compare means of non‐normal distributions.

Categorical variables were summarized as numbers and percentages. We made comparisons via analysis of chi‐square for categorical variables between males and females. Correlations between severity of COVID‐19 and underlying disease (CHD, HT, T2DM, respiratory disease, NAFLD) were analyzed in terms of the Spearman Pearson correlation coefficient. Multivariable binary logistic regression analyses were used to assess the association among age, sex, BMI, underlying comorbidity (CHD, HT, T2DM, respiratory disease, NAFLD), history of smoking, alcohol consumption and the dependent variable of severity of disease (general group and refractory group). Results of logistic regression are given as the odds ratio (OR) with the 95% confidence interval (CI). A *p* value < 0.05 was considered statistically significant using two‐sided tests. The data were analyzed by SPSS version 16.0 and Graph Pad Prism 8.0.

## 3 RESULTS

### 3.1 Baseline characteristics for different sex groups

A total of 185 patients were admitted and initially diagnosed with COVID‐19, among whom 95 (51.4%) were men. The age of all patients ranged from 18 to 92 years with a median of 41 years, furthermore, there is no differences in age between males and females (*p *= 0.914). Based on data collected, the mean BMI was higher in males (25.45 vs. 22.29, *p *< 0.001), and the mean duration from symptoms initiation to hospital admission was longer for men than that for women (6 vs. 4, *p *= 0.042). Considering previous living habits of patients, the number of patients with smoking and drinking history was significantly higher in male patients (specifically smoking: 18.9% vs. 2.2% and drinking: 28.4% vs. 6.7%, *p *< 0.001). Among all patients, 77 cases (41.6%) had one or more following comorbidities including but not limited to HT (22.7%), NAFLD (14.6%), respiratory diseases (9.2%), T2DM (7.6%), CHD (3.2%). More specifically, the percentages of males with CHD (6.3% vs. 0.0%) and NAFLD (20.0% vs. 8.9%) were significantly higher than females (*p *< 0.05) (Table 1).

**TABLE 1:** Demographics and baseline characteristics of patients infected with SARS‐CoV‐2 according to sex

Variables	All patients (*n* = 185)	Men (*n* = 95)	Women (*n* = 90)	*p* values
Age	41 (32, 57)	42 (32, 54)	40.5 (29.75, 57.25)	0.914
BMI	23.94 ± 4.41 (*n* = 132)	25.45 ± 4.40 (*n* = 69)	22.29 ± 3.81 (*n* = 63)	<0.001
From symptom to diagnosis^#^	5 (3, 8)	6 (3, 8)	4 (3, 8)	0.042
History of smoking	20 (10.80%)	18 (18.90%)	2 (2.20%)	<0.001
Alcohol consumption	33 (17.80%)	27 (28.40%)	6 (6.70%)	<0.001
Coronary heart disease	6 (3.20%)	6 (6.30%)	0 (0.00%)	0.029
Hypertension	42 (22.70%)	24 (25.30%)	18 (20.00%)	0.393
Non‐alcoholic fatty liver disease	27 (14.60%)	19 (20.00%)	8 (8.90%)	0.032
Respiratory disease	17 (9.20%)	7 (7.40%)	10 (11.10%)	0.378
Diabetes	14 (7.60%)	9 (9.50%)	5 (5.60%)	0.314
Peak temperature,°C*	38.0 (36.50, 38.50)	38.20 (37.50, 38.60)	37.65 (36.50, 38.30)	0.008
Fever ≥ 38°C	97 (52.40%)	57 (60.00%)	40 (44.40%)	0.034
HR	88 (80, 99)	89 (81, 98)	86 (78, 99)	0.620
RR	20 (17, 20)	20 (17, 21)	19 (17, 20)	0.559
SBP	128 (117, 140)	130 (121, 143)	126 (112, 137.25)	0.004
DBP	83 (75, 90)	85 (76, 91)	80 (74, 89)	0.030
O2 Saturation < 93%	19 (10.30%)	10 (10.50%)	9 (10.00%)	0.906
Headache	34 (18.40%)	16 (16.80%)	18 (20.00%)	0.579
Fever	138 (74.60%)	74 (77.90%)	64 (71.10%)	0.289
Fatigue	66 (35.70%)	36 (37.90%)	30 (33.30%)	0.517
Cough	104 (56.20%)	62 (65.30%)	42 (46.70%)	0.011
Sore throat	37 (20.00%)	21 (22.10%)	16 (17.80%)	0.462
Expectoration	55 (29.70%)	29 (30.50%)	26 (28.90%)	0.808
Shortness of breath	30 (16.20%)	13 (13.70%)	17 (18.90%)	0.337
Nausea/vomiting	10 (5.40%)	5 (5.30%)	5 (5.60%)	0.930
Diarrhea	17 (9.20%)	11 (11.60%)	6 (6.70%)	0.248
Myalgia/arthralgia	52 (28.10%)	29 (30.50%)	23 (25.60%)	0.452
Chills	36 (19.50%)	24 (25.30%)	12 (13.30%)	0.041
Signs or symptoms ≥ two	138 (74.60%)	75 (78.90%)	63 (70.00%)	0.162

*Note*: The normality of continuous variables’ distribution was tested by one‐sample Kolmogorov‐Smirnov test. Continuous variables with normal distribution were presents as mean ± standard deviation (SD); Mean of two continuous normally distributed variables was compared using independent samples Student's test; non‐normal variables were reported as median (interquartile range [IQR]). Mann‐Whitney U test and Kruskal‐Wallis test were used, respectively, to compare means of non‐normal distributions. Categorical variables were summarized as numbers and percentages. ^#^ “From symptom to diagnosis” indicates the days.* The peak temperature was measured prior to hospital admission.

### 3.2 Vital signs and laboratory parameters

All vital signs and laboratory parameters were collected on the day of hospital admission for all patients. The peak temperature measured prior to hospital admission was higher in male patients than females (38.20°C vs. 37.65°C, *p *= 0.008). Moreover, the medians of SBP, DBP in males were higher than in females (*p *< 0.05). There was no significant difference in HR and respiratory rate between male and female patients. The most common symptoms observed onset were fever (74.6%), cough (56.2%), fatigue (35.7%), expectoration (29.7%), myalgia/arthralgia (28.1%), sore throat (20.0%), and chills (19.5%). Furthermore, several less common symptoms such as headache (18.4%), diarrhea (9.2%), nausea, and vomiting (5.4%) were also observed. Of the 185 patients, 138 patients (74.6%) had two or more symptoms concurrently. Among all the listed symptoms, the percentages of patients with fever (T ≥ 38°C, 60.0% vs. 44.4%), cough (65.3% vs. 46.7%) and chills (25.3% vs. 13.3%) were significantly higher in male group than females (*p *< 0.05) (Table 1). Although leukocyte count, neutrophil count, lymphocyte count did not differ between two groups (*p *> 0.05), the platelet count was significantly lower in male group than that in female group (*p *< 0.001). However, no sex differences were observed in lymphopenia and thrombocytopenia (*p *> 0.05). In addition, there were numerous factors showed significant higher proportion in males than females including ALT, CK, CRP (*p *< 0.001, *p* = 0.006, *p *= 0.008, respectively). Of note, FIB, another acute response protein, was also higher in male patients than that in female patients (*p *= 0.033) (Table 2).

**TABLE 2:** Laboratory findings on admission to hospital of patients infected with SARS‐CoV‐2

Variables	All patients (*n* = 185)	Men (*n* = 95)	Women (*n* = 90)	*p* values
Leukocyte, ×10^9^/L	5.36 ± 2.36	5.40 ± 2.10	5.32 ± 2.60	0.805
Lymphocyte count, ×10^9^/L	1.37 ± 0.61	1.31 ± 0.57	1.44 ± 0.65	0.169
NLR^$^	2.50 (1.63, 3.58)	2.62 (1.78, 4.07)	2.41 (1.37, 3.36)	0.110
Haemoglobin, g/L	140.61 ± 17.22	149.85 ± 15.24	130.86 ± 13.41	<0.001
Lymphopenia*	123 (66.50%)	67 (70.50%)	56 (62.20%)	0.232
Thrombocytopenia^#^	39 (21.10%)	25 (26.30%)	14 (15.60%)	0.073
blood platelet count, ×10^9^/L	197 (154, 242)	176 (147, 225)	223 (172, 263.25)	<0.001
Sodium, mmol/L	139.1 (137.0, 140.6)	138.1 (136.1, 140.5)	139.9 (138.65, 141.15)	0.001
Potassium, mmol/L	3.71 (3.52, 4.00)	3.8 (3.58, 4.00)	3.7 (3.45, 3.95)	0.027
Creatinine ≥ 133, μmol/L	3 (1.60%)	1 (1.10%)	2 (2.20%)	0.613
Aspartate aminotransferase > 40 U/L (*n* = 176)	28 (15.90%)	19 (20.40%)	9 (10.80%)	0.083
Alanine aminotransferase > 40 U/L	36 (19.50%)	29 (30.50%)	7 (7.80%)	<0.001
Total bilirubin ≥ 17.1, μmol/L (*n* = 175)	22 (12.50%)	13 (14.00%)	9 (10.80%)	0.530
Albumin, g/L	42.4 (38.75, 45.45)	42.4 (38, 46.30)	42.2 (39.5, 45.2)	0.661
Creatinine kinase ≥ 200 U/L	23 (12.40%)	18 (18.90%)	5 (5.60%)	0.006
C‐reactive protein level ≥ 10 mg/L (*n* = 182)	74 (40.70%)	47 (50. 00%)	27 (30.70%)	0.008
Serum amyloid A ≥ 10 mg/L (*n* = 134)	82 (61.20%)	48 (61.60%)	34 (53.10%)	0.067
Lactose dehydrogenase ≥ 250 U/L (*n* = 173)	55 (31.80%)	34 (37.40%)	21 (25.60%)	0.097
D‐dimer ≥ 0.5 (*n* = 171)	61 (35.70%)	33 (36.30%)	28 (35.00%)	0.863
Fibrinogen, μg/mL (*n* = 184)	274 (216, 365)	306 (214, 386)	247 (219, 249)	0.033

*Note*: Continuous variables with normal distribution were present as mean ± standard deviation (SD). Mean of two continuous normally distributed variables was compared using independent samples Student's test; non‐normal variables were reported as median (interquartile range [IQR]). Mann‐Whitney U test and Kruskal‐Wallis test were used, respectively, to compare means of non‐normal distributions. Categorical variables were summarized as numbers and percentages. *Lymphopenia denoted the lymphocyte count of less than 1500 per cubic millimeter. ^#^ Thrombocytopenia denoted the platelet count of less than 150,000 per cubic millimeter; ^$^NLR, neutrophil lymphocyte ratio.

### 3.3 Chest computed tomographic images

**FIGURE 1:**
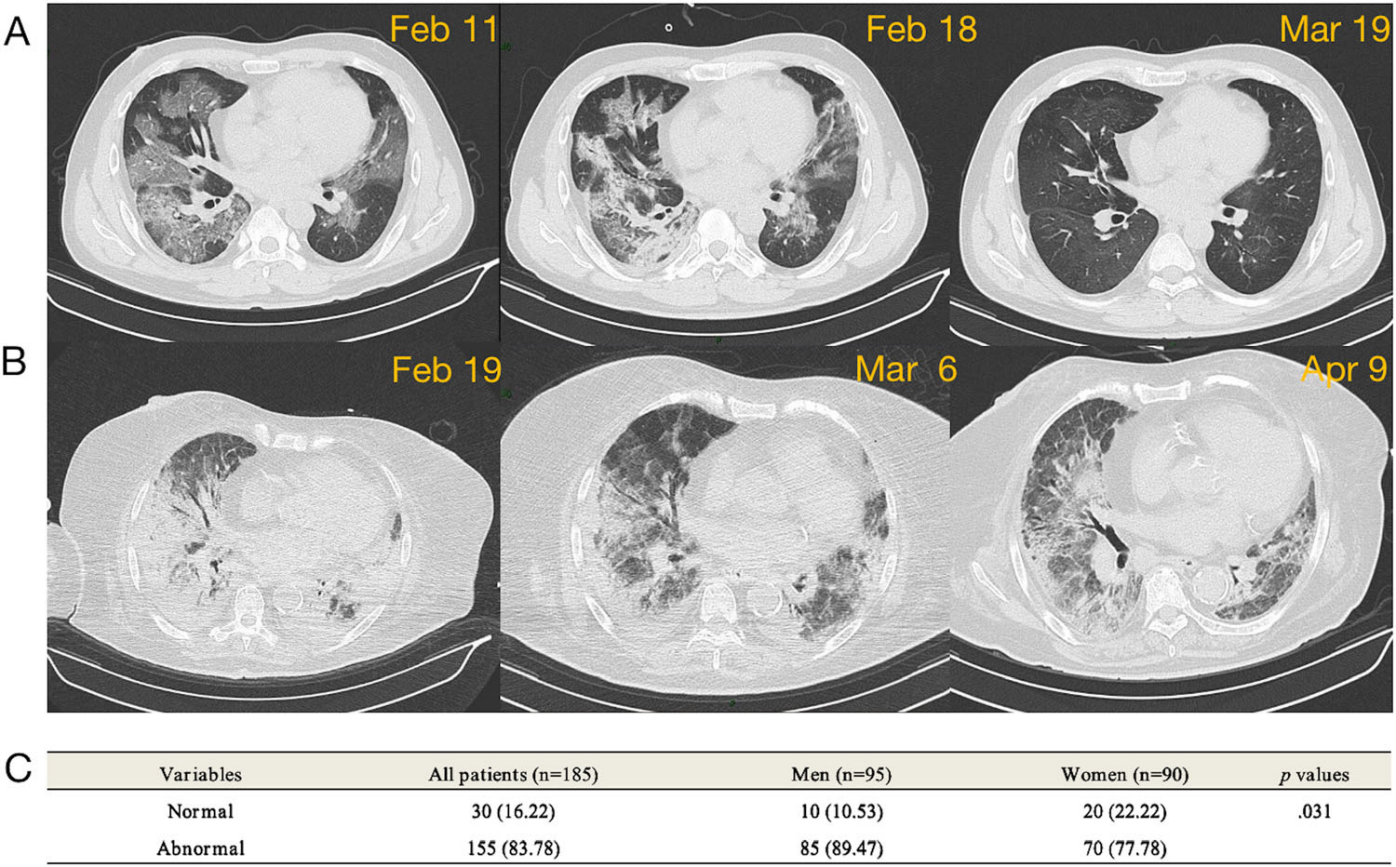
Chest computed tomographic (CT) images. (A) Chest CT images of a male patient infected with SARS‐CoV‐2 were partially absorbed after nasal high‐flow oxygen inhalation treatment. (B) Chest CT images of a female patient infected with SARS‐CoV‐2 showed patchy ground glass shadows and diffuse pulmonary fibrosis. (C) Higher percentages of CT abnormalities were discovered in male patients at the first examination after admission

We analyzed the chest CT imaging of all 185 patients at the first examination after admission. All data analyzed from CT were divided into two categories: normal and abnormal. We performed a R × C chi‐square test to compare chest CT imaging between two groups. Higher percentages of CT abnormalities were discovered in male patients (89.47% vs. 77.78%, *p *= 0.031) (Figure 1).

### 3.4 Severity assessment and clinical complications on admission

**FIGURE 2:**
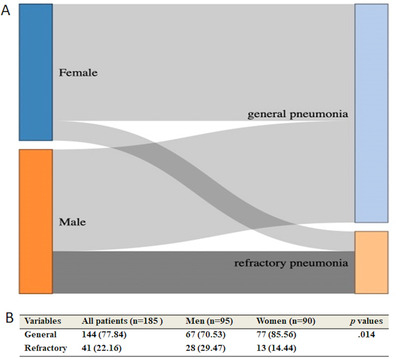
Severity of patients infected in male and female groups on admission. (A) Sankey diagram for general and refractory illness according to the clinical efficacy after hospitalization between male and female groups. (B) Refractory patients in the male group were more than that in female group

As described in the diagnostic and treatment guideline for SARS‐CoV‐2 issued by the Chinese National Health Committee, all patients were divided into general and refractory groups according to the clinical efficacy after hospitalization. By comparing the severity of disease in two groups, the rate of patients diagnosed as general and refractory illness and refractory patients in the male group were more than that in female group (*p *= 0.014) (Figure 2). There is no significant difference between males and females in complications, such as ARDS, acute kidney insufficiency (AKI) or acute exacerbation of chronic kidney insufficiency, diffuse DIC, acute myocardial injury, co‐infection and septic shock (*p* > 0.05) (Table 3).

**TABLE 3:** Clinical complications and outcome of patients infected on admission

Variables	All patients (*n* = 185)	Men (*n* = 95)	Women (*n* = 90)	*p* values
ARDS	18 (9.70%)	10 (10.50%)	8 (8.90%)	0.707
Acute renal injury	1 (0.50%)	1 (1.10%)	0 (0.00%)	>0.99
Septic shock	4 (2.20%)	3 (3.20%)	1 (1.10%)	0.339
Co‐infections	57 (30.80%)	32 (33.70%)	25 (27.80%)	0.384
Myocardial damage	6 (3.20%)	4 (4.40%)	2 (2.10%)	0.629
DIC	0	0	0	1
≥two complications	61 (33.00%)	34 (35.80%)	27 (30.00%)	0.402
Remained in hospital	72 (38.90%)	35 (36.80%)	37 (41.10%)	0.552
Recovery	96 (51.90%)	48 (50.50%)	48 (53.30%)	0.703
Mortality	2 (1.10%)	2 (2.10%)	0 (0.00%)	0.501
Readmission	15 (8.10%)	10 (10.50%)	5 (5.60%)	0.216

*Note*: The observation period of this study was from January 13 to March 19, 2020. Discharge criteria: (1) Body temperature returns to normal for more than 3 days; (2) Respiratory symptoms improved significantly; (3) Pulmonary imaging showed significant improvement of acute exudative lesions; (4) Two consecutive negative nucleic acid tests of respiratory tract specimens such as sputum, nose and throat swabs (sampling time interval of at least 24 h). Reason for readmission: reactivation of SARS‐CoV‐2 after discharge.

### 3.5 Outcomes of patients with COVID‐19

As of March 23, 2020, a total of two patients died during this period, both of whom were males. The mortality rate of COVID‐19 in this study was 1.1%, which did not differ between two groups (*p *= 0.501) (Table 3). Furthermore, there was no statistical difference in the number of patients remained in hospital, discharged, re‐admitted caused by reactivation of SARS‐CoV‐2 after discharge (*p *> 0.05) (Table 3).

### 3.6 Risk factors analysis of COVID‐19 severity

**FIGURE 3:**
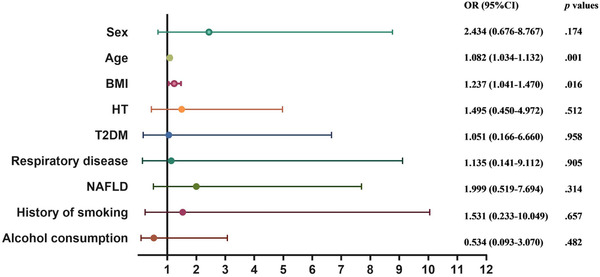
Risk factors for severity of COVID‐19. Multivariable binary logistic regression analyses were used to assess the association among age, sex, BMI, underlying comorbidity, history of smoking, alcohol consumption and the dependent variable of severity of disease (general group and refractory group). Age and BMI were considered risk factors for refractory pneumonia

Patients were classified in two groups based on severity of COVID‐19 as described previously. Correlations between severity of COVID‐19 and underlying disease (CHD, HT, T2DM, respiratory disease, and NAFLD) were analyzed in terms of the Spearman correlation coefficient, but weak correlations or no correlation were found with CHD (*p *= 0.007, *r* = 0.196), HT (*p *< 0.001, *r* = 0.270), T2DM (*p *< 0.001, *r* = 0.290), respiratory disease (*p *< 0.001, *r* = 0.281), NAFLD (*p *= 0.132, *r* = 0.111) and disease severity, respectively. Due to the limited sample size of CHD, the data could not be counted accurately and effectively in multivariate regression, only age (analyzed in quartiles, OR: 1.082; 95% CI: 1.034–1.132; *p *= 0.001) and BMI (OR: 1.237; 95% CI: 1.041–1.47; *p *= 0.016) could be considered risk factors for refractory pneumonia in multivariate regression analysis (Figure 3). We added Spearman correlation analysis between male sex and BMI and found it to be significant (*p *< 0.001, *r* = 0.486).

## 4 DISCUSSION

As reported previously, male and female may differ in susceptibility and response to certain infectious diseases. For example, men are more susceptible to certain infectious diseases, such as malaria, SARS, Middle East respiratory syndrome (MERS) and influenza than females.^4^, ^11^, ^12^ As known, infectious diseases may cause inflammatory‐mediated immune response, and the differences in immune responses between women and men may be related to sex hormones and specific X‐chromosome‐encoded genes.^13^ Many previous reports and studies have demonstrated the importance of taken sex difference into consideration during disease research. Taken all the facts into consideration, sex might play a critical role in COVID‐19 studies as well. In support of this hypothesis, many existing researches focusing on COVID‐19 have reported that there were more male patients admitted than females in the same area, other than that, the mortality and severity rates were also higher in male patients.^3^, ^14^, ^15^ All previous reports suggested possible sex effects during the progression of COVID‐19. In this study, by comparing the baseline data of male and female patients, the mean age and sex ratios of the two groups of patients were similar, which might be limited by geographical factors. However, in male patients, the proportions of smokers (18.9% vs. 2.2%), patients with CHD (6.3% vs. 0.0%) and patients with NAFLD (20.00% vs. 8.89%) were higher than those of female patients. Moreover, more male patients developed symptoms such as high fever, cough, and chills. In the related content, the abnormal CT rate in male patients is higher than that in female patient, suggesting the clinical symptoms were more sever and the pulmonary infection progresses faster in male patients. Based upon analyzed data, the refractory illness rate (including critical and severe COVID‐19) is higher in the male group than in the female group, which is consistent with the results of recent COVID‐19 studies in other regions.^16^, ^17^

Many believes that the sex differences of SARS‐CoV‐2 is related to the difference in ACE2 content in men and women, furthermore, sex is used as a strong indicator of ACE2 concentration.^18^ ACE2 exists not only in lung, but also in other types of tissue such as heart muscle, kidneys, and blood vessel walls and is particularly high in testis. It has been reported that SARS‐CoV‐2 may infect the male genitourinary system, presumably inhibiting the function of cells and reproduction. In support of this, a retrospective study of serum samples collected from 81 male patients with COVID‐19 who were admitted to Leishen Mountain Hospital, Wuhan. They found that luteinizing hormone and prolactin levels were significantly increased, while testosterone and follicle‐stimulating hormone ratios were significantly decreased in patients with COVID‐19. This might be caused by damage to the interstitial cells.^19^ The gene encoding ACE2 is located on the X chromosome. Any X chromosome‐related diseases generally showed higher infectious rate in males than in females due to the genetic chromosome differences.^20^, ^21^ As we discovered similar sex differences patterns in COVID‐19 disease research, we propose that ACE2 might be related to the sex differences in COVID‐19 between males and females as well. Kimberly E. Stelzig demonstrated that estrogen regulates the expression of SARS‐CoV‐2 receptor ACE2 in differentiated airway epithelial cells in a single female donor of NHBE cells; however more research is needed to confirm this finding.^22^ Other than the genetic contribution, different cultural and behavioral habits might act into the sex differenced in COVID‐19 as well. In this cohort, the percentage of patients who were smokers was significantly higher in male group than that in female group. Long‐term smoking might cause underlying lung diseases, which will affect lung ventilation and exacerbate lung disease progression, which played an important part in COVID‐19 progressions. Other than that, during the admission process, we found out that men intended to contradict hospital admission and thus were hospitalized longer after onset symptoms observed. This finding suggested that, compared with men, women were more willing and optimistic on seeking medical helps.^23^

Inflammatory storms have been mentioned in many studies, and our study also confirmed the inflammatory response.^24^ This study indicated that male patients had higher levels of inflammatory markers (including CRP, SAA, FIB), suggesting that bacterial infection is more common in male patients and might aggravate the disease progression.^25^, ^26^ The elevated AST and CK levels suggested that it may be related to the tissue damage mediated by virus, and the male response is more severe than female. Although the mean platelet counts in males were significantly lower than those in females, the mean platelet counts in both groups were within the normal range, and the thrombocytopenia ratios were parallel between the two groups; therefore, there was no clinical value in the decrease of platelet count between male and female groups. Correlations between severity of COVID‐19 and underlying disease (CHD, HT, T2DM, respiratory disease, NAFLD) were analyzed in terms of the Spearman correlation coefficient, but weak correlations or no correlation were found between underlying disease and disease severity respectively. This result is different from previous studies, which may be caused by low proportion of patients with underlying diseases or critically ill patients included in our study. After logistic regression analysis between severe and non‐severe cases, we found that age and BMI were independent risk factors, there was a positive correlation between male sex and BMI, which might contribute to the fact that male group had more patients with severe pneumonia and testified the research conclusions of Cai et al and Petrakis et al.^27^, ^28^ Ttorio Emanuele Bianchi conducted a meta‐analysis and found that low testosterone levels were associated with high levels of adipocytokines and inflammatory responses. Adipose tissue is a source of many inflammatory factors and may contribute to a more severe inflammatory response in elderly male obese patients.^29^ In particular, the decrease in testosterone levels in older men and the increase in pro‐inflammatory cytokines in obese patients may be particularly pronounced in older obese men, possibly exacerbating COVID‐19 progression.^30^

There were several limitations in the current study. First of all, we observed that there was a correlation between obesity and refractory pneumonia. The mechanism of obesity causing refractory pneumonia needs further research in the future. Second, Liu et al reported that the viral load of SARS‐CoV‐2 might be a useful marker for assessing disease severity and prognosis,^31^ while the viral load of SARS‐CoV‐2 was not be detected because of the emergency in progress and limited time availability. We will detect the viral load in the samples which are currently retained in the future and produce conclusions regarding to the relationship between viral load and disease characteristics and prognosis. Thirdly, this is a respective study; the findings need to be confirmed by a randomized controlled study in the future.

In this single‐center case series study, we found that age and BMI were associated with disease severity of hospitalized COVID‐19 patients in Beijing even after adjusting for other related potential confounders. Males with COVID‐19 usually had more respiratory symptoms and abnormal laboratory results, such as CRP, FIB, AST, and CK than females while they were less aware of care‐seeking than females. The findings of the current study showed that SARS‐CoV‐2 was more likely to affect older males with comorbidities. Further researches are still needed to explain the exact relationships between BMI and the severity of COVID‐19 in male patients.

## CONFLICT OF INTEREST

Authors have no conflict of interests.

## ETHICS APPROVAL AND CONSENT TO PARTICIPATE

This study was performed after approval by the Committee for Ethical Affairs of the Beijing Ditan Hospital (DTZZLX‐202009).

## AUTHOR CONTRIBUTIONS

Jing‐Jing Wang, Yun‐Juan Su, and Wen Xie conceived and designed the study. Jing‐Jing Wang, Qi Wang, and Ai‐Bin Wang extracted information and analyzed the data. Jing‐Jing Wang and Yun‐Juan Su wrote the manuscript. Ying Cao and Rui Ding reviewed the manuscript. All authors reviewed and approved the final version of the manuscript.

## AVAILABILITY OF DATA AND MATERIALS

The datasets used and/or analyzed during the current study are available from the corresponding author.
